# Behenic acid protects the testosterone cycle and prevents the sperm apoptosis and protein loss in phthalate exposure by inhibiting oxidative stress and stimulating ATPase activity

**DOI:** 10.1016/j.toxrep.2024.101845

**Published:** 2024-11-29

**Authors:** Khaled M.M. Koriem, Mayar S.R. El-Masry

**Affiliations:** a Department of Medical Physiology, Medical Research and Clinical Studies Institute, National Research Centre, El-Buhouth Street, P.O. Box. 12622, Dokki, Giza, Egypt; b Department of Pharmacology and Toxicology, Faculty of Pharmacy, Cairo University, Kasr El-Aini Street, P.O. Box.11562, Cairo, Egypt

**Keywords:** Behenic acid, Diethyl phthalate, Hypothalamus, Testis, Sperm, Spermatid

## Abstract

**Background:**

Plastic products use phthalate to enhance their flexibility, transparency, and stability, while behenic acid is a carboxylic acid with antioxidant activity.

**Objectives:**

This study evaluates whether behenic acid can protect the testosterone cycle and prevent the sperm apoptosis and protein loss in phthalate-treated male rats.

**Methods:**

There were 36 male albino rats in all, divided into six equal sets of six rats each: control, behenic acid (13 g/kg), behenic acid (26 g/kg), diethyl phthalate (10 mg/kg), behenic acid (13 g/kg) + diethyl phthalate (10 mg/kg), and behenic acid (26 g/kg) + diethyl phthalate (10 mg/kg)-treated groups. Measurements were made of serum male hormones, sex hormone-binding globulin, sodium/potassium ATPase, superoxide dismutase, glutathione, glucose-6-phosphate dehydrogenase, 3β-hydroxysteroid dehydrogenase, protein, and cholesterol in the testis, as well as malondialdehyde in the sperm, testis, and hypothalamus. Sperm monoclonal proliferating antibody Ki-67, sperm counts, motility, and abnormalities were measured.

**Results:**

Oral administration of diethyl phthalate increased malondialdehyde, serum follicle stimulating hormone, sex hormone binding globulin, luteinizing hormone, glucose-6-phosphate dehydrogenase, 3β-hydroxysteroid dehydrogenase, cholesterol, total protein, sperm abnormality, and the percentage of spermatogonia, first spermatocyte, second spermatocyte, and spermatid in the testis. Superoxide dismutase, glutathione, serum testosterone and dehydroepiandrosterone sulphate, sperm count and motility, and sodium/potassium-ATPase activity were all reduced. Additionally, all of the previously described parameters reverted to near control values after receiving two doses of behenic acid in phthalate-treated rats; a higher dose of behenic acid had a more effective effect than a lower dose.

**Conclusion:**

behenic acid can protect the testosterone cycle and prevent the sperm apoptosis and protein loss in phthalate-treated male rats by inhibiting oxidative stress and stimulating ATPase activity.

## 1 Introduction

When endocrine-disrupting chemicals (EDCs) attach to their receptors, they imitate or counteract the effects of naturally occurring hormones [1], [2]. Shampoos, clothes, toys, deodorant, cosmetics, soaps, fabrics, carpets, linens, and daily-used materials like permanent makeup and body creams are only a few examples of daily substances that contain EDCs [3], [4]. EDCs have a harmful effect on testicular function, and the sperm counts in offspring decrease over time as EDC exposure increases during pregnancy and after birth [5]. Food and agricultural packages, as well as water and air pollutants, contain EDCs. Plastic products use phthalate esters to enhance their flexibility, transparency, and stability. Phthalates are present everywhere in our modern world, where phthalates and their metabolites are found in human semen and can penetrate the skin of an individual, food, or air [6], [7]. Phthalate imitates internal hormones, binds or blocks internal hormone receptors, interferes with the metabolism of these receptors, and causes endocrine system disturbance [6], [8]. The synthesis of steroid-producing proteins, growth factor expression, and activation of germ cell death are affected by phthalate exposure [9], [10], [11], [12]. Phthalate increases lipid peroxidation and decreases the glutathione defense network, which results in ferroptosis [13]. Phthalate produced disturbance of Sertoli and Spermatogonia cells, as well as degradation of the blood-testis barrier integrity [14]. Phthalate caused cytoskeletal degradation and cell cycle arrest in Sertoli cells and impacted the metabolism of testosterone in Leydig cells. In all testicular cells, phthalate acts through two mechanisms: increased oxidative stress and excessive apoptosis [15]. Following exposure to phthalates, spermatogenesis was disrupted, and damage to the seminiferous epithelium was noted. In the meantime, pyroptosis and damage caused by oxidative stress were triggered. Thus, in rat testes, phthalate caused an imbalance in pyroptosis and mitophagy mediated by reactive oxygen species [16].

Secondary metabolites constitute a vast range of molecules coming from all different biological sources; they are of great interest due to their significant involvement in the prevention and treatment of numerous human illnesses, including male dysfunction. Behenic acid (also known as docosanoic acid) is a carboxylic acid, and its molecular formula is C_21_H_43_COOH. Behenic acid occurs in many oils, such as ben oil, peanut oil, and rapeseed oil [17]. One ton of peanut contains 5.9 kg of behenic acid [18]. Behenic acid is the major constituent in *Moringa oleifera* seeds [19]. Behenic acid is safe and without any toxic effect [20], shows antimicrobial action [21], has an anti-tumor effect [22], [23], and is used as a medicinal food for diabetes mellitus [24]. Higher behenic acid levels improved human aging and decreased the risk of type 2 diabetes, coronary heart disease, atrial fibrillation, abrupt heart failure, and death [25]. There is an inverse correlation between behenic acid in the cerebrospinal fluid and postpartum depression in women (depressive episodes following birth) [26]. Behenic acid decreased the incidence of type 2 diabetes because it significantly affected β-cell function and insulin sensitivity [27]. Behenic acid has a protective role in acute stroke because it has an inverse correlation with acute ischemic stroke in hypertension patients [28]. Behenic acid has potent multi-target antibacterial activity. It works antagonistically against bacterial infections. Its activity in various tissues depends on the inhibition of catalase peroxidase, adenylosuccinate synthetase, and pyridoxine 5′-phosphate synthase [29].

The research gap is that humans cannot avoid phthalate exposure due to its incorporation into plastic products; therefore, the hypothesis guiding this study is to find a natural substance that is inexpensive, safe, and does not harm sperm that protects the testosterone cycle and prevents the sperm apoptosis and protein loss in phthalate-exposed male rats. Thus, the goal of the study is to evaluate behenic acid's capacity to safeguard the testosterone cycle and to prevent sperm apoptosis and protein loss in phthalate-treated male rats.

## 2 Materials and methods

### 2.1 Materials

Both behenic acid (Product No. 216941; CAS No. 112–85–6; purity: 99 %; Molecular Weight: 340.58) and diethyl phthalate (CAS No. 84–66–2; purity: 99.5 %; Product No. 524972) were acquired from Sigma-Aldrich in the US. The kits for testosterone (Ts), luteinizing hormone (LH), follicle stimulating hormone (FSH), and dehydroepiandrosterone sulfate (DHEA-SO4) were purchased from BioSource Co., Nivelles, Belgium. We bought kits for 3β-hydroxysteroid dehydrogenase (3βHSD), glucose-6-phosphate dehydrogenase (G6PD), and sex hormone-binding globulin (SHBG) from IBL Company in Hamburg, Germany. Furthermore, kits for testing glutathione (GSH), malondialdehyde (MDA), cholesterol, total protein, and superoxide dismutase (SOD) were purchased from the Randox Company in Crumlin, UK. The monoclonal proliferative antibody, Ki-67, was supplied by Zymed Laboratories Incorporation (San Francisco, California, USA). All of the analytical-grade compounds used in the study were acquired from Egyptian branches.

### 2.2 Animals

Adult male *Sprague Dawley* albino rats weighing 140–145 g at 10 weeks of age were obtained from the National Research Center's animal house colony in Egypt. They were housed in steel cages with free access to tap water and a rat diet. Standard conditions for the animals' housing included a 12-hour light/dark cycle, a standard temperature range of 26°C to 30°C, and a humidity range of 40–70 %. The Institutional Animal Ethical Committee (IAEC) of the National Regulations on the Animal Welfare of the National Research Center, Egypt, granted approval for the study with approval number 13150452. The care and treatment of laboratory animals during research is governed by National Institutes of Health (NIH) document no. 85:23, which was updated in 1985.

### 2.3 Study design

In the first pilot study, five doses of behenic acid (1 g/kg, 4 g/kg, 7 g/kg, 10 g/kg, and 13 g/kg) were evaluated first in accordance with the earlier study [30]. Rats treated with phthalate (6 rats per group) received each of these doses orally for two weeks. The blood was taken from these after two weeks of behenic acid oral treatment in order to measure the levels of the male hormones (Ts, LH, FSH, DHEA-SO4, and SHBG) in their serum. The best dose recorded to detect the serum male hormone measurement was 13 g/kg of behenic acid.

Based on the findings of the first pilot study, the second study included 36 male animals divided into 6 equal groups, each containing 6 rats: (1) Control: rats in the control group received 1 ml of distilled water orally once daily for a duration of one month. (2) Behenic acid (13 g/kg)-treated group: rats received 1 ml of behenic acid (1 g/kg) orally once day for a month. (3) Behenic acid (26 g/kg)-treated group: rats received 2 ml (26 g/kg) of behenic acid orally once daily for a month. (4) Diethyl phthalate-treated group: rats received 1 ml (10 mg/kg) of diethyl phthalate orally once daily for a month [31]. (5) Behenic acid (13 g/kg) + diethyl phthalate (10 mg/kg) treated group: rats received an oral dose of 1 ml of behenic acid (13 g/kg) and, an hour later, 1 ml of diethyl phthalate (10 mg/kg) for a duration of one month. (6) Group treated with behenic acid (26 g/kg) first then diethyl phthalate (10 mg/kg): rats receiving an oral dose of 2 ml (26 g/kg) of behenic acid, rats then orally intake of 1 ml (10 mg/kg) of diethyl phthalate once daily for a month.

All rats were monitored for any unusual symptoms during the entire experiment, including rat skin patches, convulsions, hair loss, or animal death.

### 2.4 Obtaining a blood sample

Following an overnight fast, the animals were beheaded at the conclusion of a one-month experimental research. Blood samples were drawn via jugular vein punctures using non-heparinized tubes and thereafter stored in ice. Centrifugation was used to obtain blood serum at 3000 g for 10 minutes at −20 °C. For additional biochemical analysis, the obtained blood serum was kept at −80 °C.

### 2.5 Preparation of testis and hypothalamic tissues

Animals were decapitated and then dissected. After being obtained, the left testis and hypothalamus were cleansed in saline, dried on two filter papers, and then cut into small pieces for homogenization before being frozen at −80 °C for additional analysis. The following is a summary of the procedure: 0.5 g of testis and hypothalamus tissues were dissolved in 2.5 ml of Tris buffer solution and then homogenized in a homogenizer (speed = 2500 g [32] for 5 minutes for hypothalamus while for 15 minutes for testis) with an ice bath. To determine the biochemical assays, the hypothalamic and testicular supernatants were separated at −40 °C.

### 2.6 An examination of sperm

The previously established procedure was used to collect sperm [33]. In summary, the epididymides of both testes were cut free, extracted, and stored in saline at 37 °C. Three deep cuts were made to the proximal and distal cauda of each epididymis. Following five minutes of incubation at 37°C, the tissue was removed, and the sperm suspensions were then gently mixed and kept at 37°C.

#### 2.6.1 Calculation of total number of sperm

The sperm suspension aliquots were diluted 100 times with fresh medium, and the Neubauer chamber [34] method was used to count the total number of sperm.

#### 2.6.2 Motility of the sperm

The aliquots of sperm suspensions were examined under a microscope. Sperm motility was evaluated by comparing the quantity of sperm to the total number of sperm.

#### 2.6.3 Abnormalities of the sperm

Smears were fixed with alcohol, then stained with Eosin-Y, and inspected for morphological abnormalities to detect the sperm abnormalities. Following a previous methodology [34], the percentages of aberrant sperm in three fields were determined.

### 2.7 Detection of antibody monoclonal proliferating index (Ki-67) in testis

In both normal and aberrant testicular tissues, Ki-67 is a nuclear antigen that forms in the nucleus during mitosis. Proteins are what keep the cell cycle going. This test was designed to evaluate the testis spermatogenesis process's activity. Immediately after rat death, the testis and epididymis of each rat were removed, cleaned with saline using a caudal puncture, and then immersed in saline. Sperm were pressed to leave the epididymal tubules by keeping the epididymus in an incubator at 37 °C for 30 minutes. The sperm suspension was used in aliquots after epididymal fragments were removed from the previously described preparation.

### 2.8 Determination of serum and testicular biochemistry

According to Maruyama et al. [35], the serum Ts was computed. The Knobil method was used to calculate serum LH [36]. The serum FSH was estimated using the Odell et al. [37] technique. The De-Peretti and Forest [38] method was used to test serum DHEA-SO4. The serum SHBG assessment method was derived from the Selby [39] method. The Chan et al. [40] approach was used to determine testicular G6PD. The Talalay [41] method was used to determine the testicular 3βHSD. Using the Lowry et al. [42] method, the total protein in the testicles was measured. Testicular cholesterol was computed using the Kim and Goldnerg [43] approach. According to Nishikimi et al. [44], Prins and Loose [45], and Ohkawa et al. [46] procedures, the antioxidant parameters SOD, GSH, and MDA in the testis, sperm, and hypothalamus were determined.

### 2.9 Assessment of sodium/potassium-ATPase in the testis, sperm, and hypothalamus

The activity of sodium/potassium ATPase was assessed in accordance with Gamaro et al. [47]. A solution including KCl (20 mM), MgCl2 (5 mM), NaCl (80 mM), Tris HCl (50 mM, pH 7.4), and ATP disodium salt (3 mM) was produced in order to carry out this procedure. This solution was then treated with 50 µL of the homogenate of either the testis, hypothalamus, or sperm for 10 minutes at 37 °C. The aforementioned solution was then mixed with 50 µL of a trichloroacetic acid solution. The solution was then centrifuged at 3000 rpm for five minutes. A solution of 250 µL ascorbic acid, 500 µL trichloroacetic acid, and 250 µL ammonium molybdate was mixed with 1 ml of the supernatant. A spectrophotometer set to 680 nm was used to measure the color that was obtained.

### 2.10 Sperm DNA apoptosis determination

Agarose gel electrophoresis was used to analyze sperm DNA fragmentation. In accordance with previous research [48], DNA was obtained. To dissolve the DNA, a pH 8.0 Tris-EDTA buffer was utilized. Using a tris-boric acid-EDTA buffer (pH 8.3), the DNA samples were electrophoresed on a 0.7 % agarose gel for four hours at 40 volts. Ethidium bromide staining was followed by the observation of DNA damage. The bands (Bio-Rad Laboratory, 6000 James Watson Dr., lot number: 10–0082 0100 Sig 1109, version number: 10016027 Rev C US/EG) were photographed.

### 2.11 Chromatography method

#### 2.11.1 Preparing a sample for analysis using gas chromatography-mass spectroscopy (GC–MS) for sperm protein detection

The authors added a 1 ml homogenate of the sperms to a 2 ml solution containing a mixture of methyl alcohol, chloroform, and water through a 2:5:2 vol ratio. As internal standards, 10 ml of ribitol and 10 ml of this solution were added with alanine-d_4_, and it was centrifuged for 10 minutes at 12,300 g. The supernatant was collected in a volume of 10 ml and dried at 37 °C using nitrogen gas. After being suspended in 5 ml of methoxyamine hydrochloride solution in pyridine for two hours, this solution was added to 50 ml of N,O-bistrimethylsilyl trifluoroacetamide and incubated for four hours at 37 °C. The sample was left to dry at 37 °C for a full day.

#### 2.11.2 GC–MS method for sperm protein detection

For the GC-MS approach, a mass-selective device and a Hewlett-Packard GC 6890 apparatus were utilized. This application was operated using the Enhanced ChemStation program. The GC-MS experiment employed an Agilent HP-5MS UI (30.0 × 3.25 mm) capillary column coated with 5 % diphenyl and 95 % dimethylpolysiloxane. Helium gas was used as the carrier gas, and the injection temperature was set at 250 °C and 1.0 ml/min. The temperature in the column increased by 10 °C every minute for five minutes, reaching 280 °C after three minutes at 80 °C. The ion source, MS quadruple, and MS transfer line were all configured at 230, 150, and 280 °C, respectively.

### 2.12 Statistical evaluation

The findings were presented as mean ± standard error of the mean (SEM) in the study. Prior to doing an analysis of variance (ANOVA), the data were examined to ensure that they followed the Gaussian (normal) distribution. ANOVA, or one-way analysis of variance test, was applied. The SPSS 13 program was used. In *post*-*hoc* analysis, the Fisher least significant difference (FLSD) test was used to determine that *P*-values of 0.05 were significant across all treatment groups.

## 3 Results

### 3.1 Male organs weights of all groups

Rats treated with diethyl phthalate had significantly decreased male reproductive organs (prostate, seminal vesicle, vasa differentia, testes, and epididymis) when compared to these organs in the control group (Table 1). Two oral doses of behenic acid administration increased the rats' male organ weight to nearly control levels. Compared to the lower dose, the higher dose of behenic acid was more active.

**Table 1:** Effect of behenic acid on male organs weights of all treated groups at the end of the experimental study.

**Parameters**	**Control**	**Beh.** **(13 mg/kg)**	**Beh. (26 mg/kg)**	**Ph. rats** (10 mg/kg)	**Beh. (13 g/kg) + Ph. rats**	**Beh. (26 g/kg) + Ph. rats**
**Testes (g)**	2.13 ± 0.08	2.12 ± 0.06	2.14 ± 0.07	1.24 ± 0.03^b^	1.68 ± 0.06^c^	2.11 ± 0.07^d^
**Epidydemis (g)**	1.25 ± 0.06	1.24 ± 0.04	1.26 ± 0.05	0.60 ± 0.05^b^	0.92 ± 0.06^c^	1.23 ± 0.04^d^
**Sem. Ves. (g)**	0.41 ± 0.04	0.40 ± 0.05	0.42 ± 0.03	0.16 ± 0.02^b^	0.28 ± 0.05^c^	0.39 ± 0.06^d^
**Prostate (g)**	0.35 ± 0.05	0.36 ± 0.07	0.34 ± 0.06	0.20 ± 0.03^b^	0.26 ± 0.06^c^	0.32 ± 0.05^d^
**Vas. diff. (g)**	0.80 ± 0.07	0.79 ± 0.06	0.81 ± 0.04	0.35 ± 0.05^b^	0.57 ± 0.04^c^	0.78 ± 0.07^d^

^a^Significant difference (*P* ≤ 0.05) from the control.

There were six rats in each group. The data are shown as mean ± SEM.

Beh.: Behenic acid. Ph. rats: Phthalate-treated rats. Sem. Ves.: Seminal vesicle. Vas. diff.: Vasa differentia.

b Highly significant difference (*P* ≤ 0.01) from the control.

c Significant difference (*P* ≤ 0.05) from phthalate-treated rats.

d Highly significant difference (*P* ≤ 0.01) from phthalate-treated rats.

### 3.2 Antioxidant levels in the sperm, testis, and hypothalamus

The antioxidant levels of the testes, sperm, and hypothalamus of rats exposed to diethyl phthalate are shown to be protected by behenic acid, as appeared in Table 2. MDA levels in the testis, hypothalamus, and sperm of the phthalate-treated rats were higher than those of the control group. Rats treated with diethyl phthalates had lower levels of SOD and GSH than the control group. However, following two oral doses of behenic acid, the antioxidant levels in the phthalate-treated rats returned to normal, with the higher dose of behenic acid having a better effect than the lower dose. In addition, rats were administered only behenic acid orally during the study; the results exhibited no effect on the antioxidant levels in their sperm, testes, or hypothalamus.

**Table 2:** Protective effect of behenic acid on antioxidant levels of hypothalamus, testis, and sperm of phthalate-treated rats.

**Parameters**	**Control**	**Beh.** **(13 mg/kg)**	**Beh. (26 mg/kg)**	**Ph. rats** (10 mg/kg)	**Beh. (13 g/kg) + Ph. rats**	**Beh. (26 g/kg) + Ph. rats**
**Hypothalamus**						
**SOD (U/g)**	160.2 ± 21.7	159.5 ± 22.3	161.4 ± 21.5	87.6 ± 19.4^b^	122.5 ± 21.4^c^	158.4 ± 20.6^d^
**GSH (mg/g)**	0.51 ± 0.06	0.50 ± 0.05	0.52 ± 0.07	0.34 ± 0.04^a^	0.42 ± 0.06^c^	0.49 ± 0.05^d^
**MDA (nmol /g)**	245.2 ± 32. 5	246.1 ± 30.8	244.8 ± 31.7	350.6 ± 28.7^b^	298.5 ± 32.4^c^	246.7 ± 31.9^d^
**Testis**						
**SOD (U/g)**	139.5 ± 19.6	138.7 ± 18.6	139.5 ± 20.3	78.9 ± 16.5^b^	109.4 ± 19.6^c^	139.5 ± 19.6^d^
**GSH (mg/g)**	161.4 ± 22.5	159.8 ± 21.4	162.1 ± 20.7	135.6 ± 19.5^a^	148.5 ± 21.7^c^	161.4 ± 22.5^d^
**MDA (nmol /g)**	49.8 ± 8.9	48.6 ± 8.5	50.1 ± 7.8	70.9 ± 8.1^b^	60.4 ± 7.9^c^	49.8 ± 8.9^d^
**Sperm**						
**SOD (U/g)**	154.6 ± 19.7	155.1 ± 18.7	153.8 ± 19.2	85.7 ± 18.4^b^	119.3 ± 19.4^c^	152.8 ± 19.7^d^
**GSH (mg/g)**	0.30 ± 0.04	0.29 ± 0.05	0.31 ± 0.04	0.20 ± 0.03^a^	0.24 ± 0.04^c^	0.28 ± 0.06^d^
**MDA (nmol /g)**	472.5 ± 43.1	471.8 ± 41.8	473.2 ± 42.8	561.4 ± 38.6^b^	517.5 ± 40.9^c^	473.6 ± 41.5^d^

There were six rats in each group. The data are shown as mean ± SEM.

Beh.: Behenic acid. Ph. rats: Phthalate (10 mg/kg)-treated rats. Beh. (13 g/kg) + Ph. rats: Behenic acid (13 g/kg) + Phthalate (10 mg/kg)-treated rats. Beh. (26 g/kg) + Ph. rats: Behenic acid (26 g/kg) + Phthalate (10 mg/kg)-treated rats.

a Significant difference (*P* ≤ 0.05) from the control.

b Highly significant difference (*P* ≤ 0.01) from the control.

c Significant difference (*P* ≤ 0.05) from phthalate-treated rats.

d Highly significant difference (*P* ≤ 0.01) from phthalate-treated rats.

### 3.3 Testicular G6PD, 3BHSD, cholesterol, and total protein levels and serum male hormones

Table 3 displays the protective effect of behenic acid on serum male hormones in rats exposed to diethyl phthalate. Based on these data, it can be said that oral diethyl phthalate treatment raised serum levels of LH, FSH, and SHBG in rats treated with diethyl phthalate while decreasing serum levels of Ts and DHEA-SO_4_. However, when two oral doses of behenic acid were given to rats treated with diethyl phthalate, the serum levels of male hormones were almost brought back to control levels; the larger dose of behenic acid was more effective than the lower one. Furthermore, rats who received only oral behenic acid during the study's experimental period did not exhibit any changes in their serum male hormone levels.

**Table 3:** Protective effect of behenic acid on serum male hormones and testicular G6PD, 3βHSD, cholesterol, and total protein of phthalate-treated rats.

**Parameters**	**Control**	**Beh. (13 g/kg)**	**Beh. (26 g/kg)**	**Ph. rats** (10 mg/kg)	**Beh. (13 g/kg) + Ph. rats**	**Beh. (26 g/kg) + Ph. rats**
**Ser. Ts (ng/ml)**	5.98 ± 0.42	5.96 ± 0.39	5.99 ± 0.49	3.14 ± 0.49^a^	4.55 ± 0.37^c^	5.95 ± 0.42^d^
**Ser. LH mIU/ml)**	18.27 ± 1.85	18.30 ± 1.76	18.25 ± 1.87	36.41 ± 2.21^b^	25.74 ± 1.69^c^	18.19 ± 1.85^d^
**Ser. FSH (mIU/ml)**	1.05 ± 0.13	1.07 ± 0.12	1.06 ± 0.10	2.32 ± 0.22^b^	1.68 ± 0.15^c^	1.03 ± 0.13^d^
**Ser. DHEA-SO**_**4**_**(µg/dl)**	197.5 ± 23.2	197.6 ± 24.8	196.8 ± 21.7	164.3 ± 17.6^a^	175.1 ± 19.8^c^	195.9 ± 23.2^d^
**Ser. SHBG (nmol/L)**	6.65 ± 0.49	6.67 ± 0.58	6.64 ± 0.51	8.54 ± 0.83^a^	7.83 ± 0.69^c^	6.59 ± 0.48^d^
**Testis G6PD (U/g)**	11.92 ± 0.75	11.90 ± 0.81	11.94 ± 0.96	7.64 ± 0.68^a^	9.76 ± 0.92^c^	11.89 ± 0.64^d^
**Testis 3βHSD (U/g)**	4.46 ± 0.83	4.45 ± 0.75	4.47 ± 0.68	2.15 ± 0.36^b^	3.29 ± 0.58^c^	4.43 ± 0.87^d^
**Testis cholesterol (mg/g)**	130.7 ± 8.23	131.4 ± 7.51	132.1 ± 8.14	75.9 ± 8.46^b^	102.4 ± 7.81^c^	128.9 ± 7.63^d^
**Testis total protein (mg/g)**	290.6 ± 15.32	292.3 ± 14.72	291.5 ± 15.29	256.8 ± 14.84^a^	273.4 ± 15.39^c^	289.7 ± 14.50^d^

There were six rats in each group. The data are shown as mean ± SEM.

Ser. Ts: Serum testosterone. Ser. LH: Serum luteinizing hormone. Ser. FSH: Serum follicle stimulating hormone. Ser. DHEA-SO_4_: Serum dehydroepiandrosterone sulfate. Ser. SHBG: Serum sex hormone binding globulin. G6PD: Glucose-6-phosphatedehydrogenase. 3βHSD: 3β-hydroxysteroid dehydrogenase. Beh.: Behenic acid. Ph. rats: Phthalate (10 mg/kg)-treated rats. Beh. (13 g/kg) + Ph. rats: Behenic acid (13 g/kg) + Phthalate (10 mg/kg)-treated rats. Beh. (26 g/kg) + Ph. rats: Behenic acid (26 g/kg) + Phthalate (10 mg/kg)-treated rats.

a Significant difference (*P* ≤ 0.05) from the control.

b Highly significant difference (*P* ≤ 0.01) from the control.

c Significant difference (*P* ≤ 0.05) from phthalate-treated rats.

d Highly significant difference (*P* ≤ 0.01) from phthalate-treated rats.

In rats treated with diethyl phthalate, Table 3 illustrates how behenic acid protects testis G6PD, 3βHSD, cholesterol, and total protein. The information in this table shows that rats treated with diethyl phthalate had lower levels of G6PD, 3βHSD, cholesterol, and total protein than the control group. Furthermore, the aforementioned parameters were forced to control levels by two oral doses of behenic acid given to rats treated with diethyl phthalate; the higher dose of behenic acid had a better effect than the lower dose. Furthermore, rats were given only behenic acid orally during the study's experimental period, and the previously described parameters remained unchanged.

### 3.4 Outcomes of sperm motility, count, and proliferative index in the testis

Table 4 shows how behenic acid protected sperm motility and count while preventing sperm abnormalities in rats treated with diethyl phthalate. This table shows that in rats treated with diethyl phthalate, oral diethyl phthalate treatment decreased sperm motility and count while increasing sperm abnormalities. Additionally, sperm abnormalities, motility, and count were almost returned to control in rats treated with diethyl phthalate after receiving two oral doses of behenic acid; the higher dose of behenic acid proved to be more beneficial than the lower dose. Furthermore, rats given oral behenic acid alone during the study's experimental period displayed no alterations in sperm motility, count, or abnormalities.

**Table 4:** Protective effect of behenic acid on sperm count, motility, and abnormality, as well as, proliferative index (Ki-67) in testis of phthalate-treated rats.

**Parameters**	**Control**	**Beh. (13 g/kg)**	**Beh. (26 g/kg)**	**Ph. rats** (10 mg/kg)	**Beh. (13 g/kg) + Ph. rats**	**Beh. (26 g/kg) + Ph. rats**
**Sp. Count (×10**^**6**^**/ml)**	185 ± 19.64	184 ± 18.75	186 ± 19.28	94 ± 18.56^b^	139 ± 18.74^c^	183 ± 19.83^d^
**Sp. Motility (%)**	86 ± 10.54	85 ± 9.63	87 ± 10.82	67 ± 9.71^a^	76 ± 10.58^c^	84 ± 10.23
**Sp. Abnormality (%)**	7.9 ± 0.58	7.8 ± 0.61	8.0 ± 0.70	9.8 ± 0.49^a^	8.7 ± 0.63^c^	7.6 ± 0.57^d^
**Spermatogonia**	6.85 ± 0.72	6.84 ± 0.59	6.86 ± 0.61	14.36 ± 0.59^b^	10.59 ± 0.73^c^	6.83 ± 0.69^d^
**1**^**st**^**spermatocyte**	13.7 ± 0.84	13.6 ± 0.79	13.8 ± 0.92	20.6 ± 0.79^a^	17.1 ± 0.64^c^	13.5 ± 0.58^d^
**2**^**nd**^**spermatocyte**	17.6 ± 0.92	18.1 ± 0.85	17.5 ± 0.79	26.4 ± 0.68^a^	21.9 ± 0.86^c^	17.4 ± 0.78^d^
**Spermatid**	0.0 ± 0.0	0.0 ± 0.0	0.0 ± 0.0	5.3 ± 0.06	2.7 ± 0.04^c^	0.2 ± 0.01^d^

Proliferative index (Ki-67) includes spermatogonia, 1st spermatocyte, 2nd spermatocyte, and spermatid. There were six rats in each group. The data are shown as mean ± SEM.

Beh.: Behenic acid. Ph. rats: Phthalate (10 mg/kg)-treated rats. Beh. (13 g/kg) + Ph. rats: Behenic acid (13 g/kg) + Phthalate (10 mg/kg)-treated rats. Beh. (26 g/kg) + Ph. rats: Behenic acid (26 g/kg) + Phthalate (10 mg/kg)-treated rats. Sp. Count: Sperm Count. Sp. Motility: Sperm Motility. Sp. Abnormality: Sperm Abnormality.

a Significant difference (*P* ≤ 0.05) from the control.

b Highly significant difference (*P* ≤ 0.01) from the control.

c Significant difference (*P* ≤ 0.05) from phthalate-treated rats.

d Highly significant difference (*P* ≤ 0.01) from phthalate-treated rats.

Table 4 displays the impact of behenic acid on the proliferative index in the testis of rats exposed to diethyl phthalate. According to the results in this table, the diethyl phthalate group's testis had a higher percentage of spermatogonia, first spermatocytes, second spermatocytes, and spermatids than the control group. Furthermore, following two doses of behenic acid administration, the aforementioned parameters in the testis of rats exposed to diethyl phthalate were brought back to levels that were comparable to control values. The impact of the higher behenic acid dose was better than that of the lower dose. Furthermore, rats given oral behenic acid alone during the study's experimental period displayed no alterations in their spermatid cells, first or second spermatocytes, or spermatocytes.

### 3.5 Results of sodium/potassium-ATPase in the testis, hypothalamus, and sperm

**Fig. 1:**
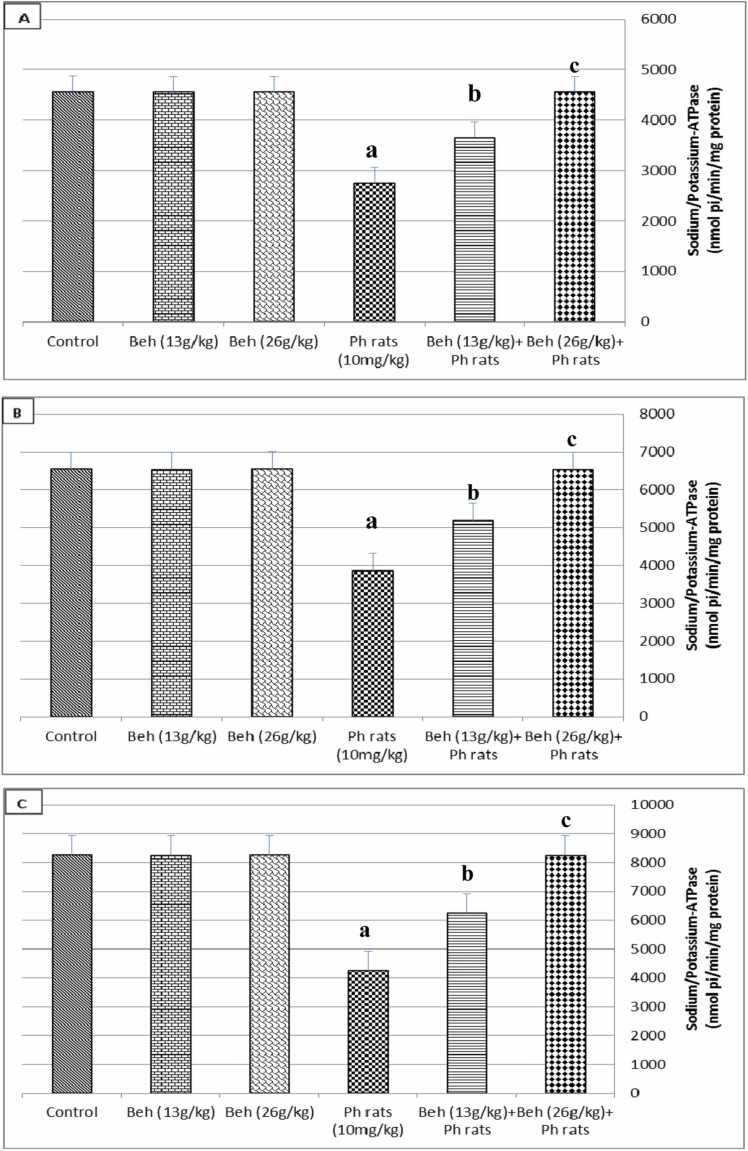
Hypothalamus, testis, and sperm sodium/potassium-ATPase activity of all groups. The sodium/potassium ATPase activity (nmol Pi/min/mg protein) is shown in this figure. in hypothalamus (A), testis (B), and sperm (C). There were six rats in each group. The data are shown as mean ± SEM. ^a^Significant difference (*P* ≤ 0.05) from the control. ^b^Highly significant difference (*P* ≤ 0.01) from the control. ^c^Significant difference (*P* ≤ 0.05) from phthalate-treated rats. ^d^Highly significant difference (*P* ≤ 0.01) from phthalate-treated rats. Beh.: Behenic acid. Ph. rats: Phthalate (10 mg/kg)-treated rats. Beh. (13 g/kg) + Ph. rats: Behenic acid (13 g/kg) + Phthalate (10 mg/kg)-treated rats. Beh. (26 g/kg) + Ph. rats: Behenic acid (26 g/kg) + Phthalate (10 mg/kg)-treated rats.

The effect of behenic acid on sodium/potassium ATPase activity in the testis, sperm, and hypothalamus of rats treated with diethyl phthalate is shown in Fig. 1. This figure demonstrates that oral treatment of diethyl phthalate dramatically decreased (*P* ≤ 0.01) sodium/potassium ATPase activity in the sperm, testis, and hypothalamus when compared to the control group. However, this activity was raised to a level that was almost equivalent to the control value when behenic acid (13 g/kg and 26 g/kg) was administered orally to rats treated with diethyl phthalate. The impact of behenic acid was dose-dependent. Rats were also given behenic acid orally alone during the research; however, this had no influence on their ability to do these parameters.

### 3.6 Sperm DNA apoptosis results

**Fig. 2:**
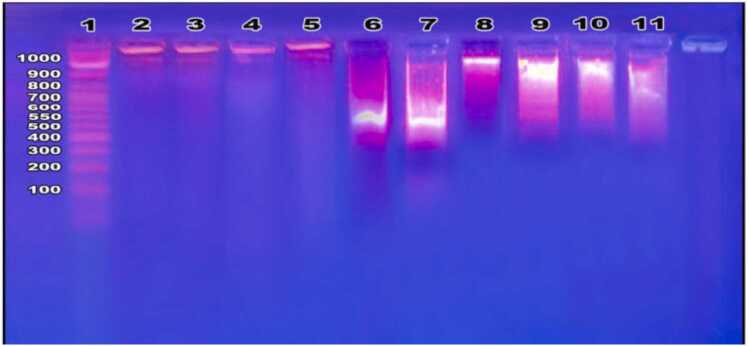
Sperm DNA fragmentation. Sperm DNA electrophoresis where:-Lane 1: DNA ladder. Lanes 2& 3: Control group. Lane 4: Behenic acid (13 g/kg) in rats. Lane 5: Behenic acid (26 g/kg) in rats. Lanes 6 and 7: Diethyl phthalate (10 mg/kg)-treated group. Lanes 8 and 9: Behenic acid (13 g/kg) + Phthalate (10 mg/kg)-treated rats. Lanes 10 and 11: Behenic acid (26 g/kg) + Phthalate (10 mg/kg)-treated rats.

Fig. 2 illustrates how behenic acid prevented DNA damage in the sperm of diethyl phthalate-treated rats. Rats given diethyl phthalate showed fragmentation of their sperm DNA in contrast to the control group. While two doses of behenic acid in normal rats did not alter sperm DNA electrophoretic patterns in the control group, behenic acid (13 g/kg and 26 g/kg) treatment dramatically reduced sperm DNA damage in diethyl phthalate-treated animals.

### 3.7 Sperm protein detection by GC-MS results

**Fig. 3:**
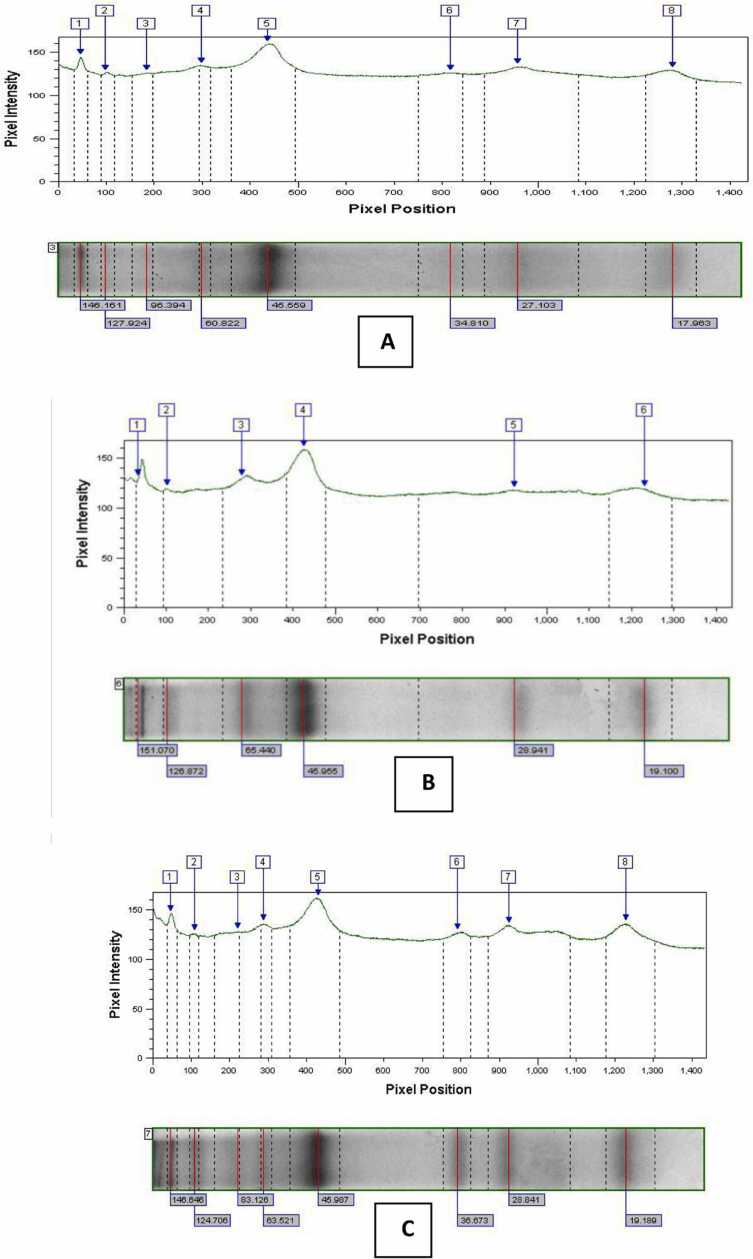
Sperm protein detection by gas chromatography-mass spectra (GC-MS). This figure shows the sperm protein detection by GC-MS method. (A) reveals the sperm of control group has 8 proteins; 1, albumin; 2, globulin; 3, prothrombin; 4, fibrinogen; 5, transferrin; 6, α2-macroglobulin, 7, α1-fetoprotein, and 8, β-2 microglobulin. (B) shows the sperm of phthalate (10 mg/kg)-treated group has 6 proteins; 1, albumin; 2, globulin; 3, prothrombin; 4, fibrinogen; 5, transferrin; and 6, α2-macroglobulin. (C) exhibits the sperm of behenic acid (26 g/kg) + phthalate (10 mg/kg)-treated rats -treated group has 8 proteins; 1, albumin; 2, globulin; 3, prothrombin; 4, fibrinogen; 5, transferrin; 6, α2-macroglobulin, 7, α1-fetoprotein, and 8, β-2 microglobulin.

Fig. 3 shows the sperm protein detection by the GC-MS method. Fig. 3A reveals the sperm of the control group, and 8 proteins were exhibited: (1) albumin; (2) globulin; (3) prothrombin; (4) fibrinogen; (5) transferrin; (6) α2-macroglobulin; (7) α1-fetoprotein; and (8) β-2 microglobulin. Fig. 3B shows the sperm of the phthalate (10 mg/kg)-treated group, and 6 proteins occurred: (1) albumin; (2) globulin; (3) prothrombin; (4) fibrinogen; (5) transferrin; and (6) α2-macroglobulin. Fig. 3C exhibits the sperm of behenic acid (26 g/kg) + phthalate (10 mg/kg). -treated rats, and 8 proteins were found: (1) albumin; (2) globulin; (3) prothrombin; (4) fibrinogen; (5) transferrin; (6) α2-macroglobulin; (7) α1-fetoprotein; and (8) β-2 microglobulin.

## 4 Discussion

The present study found that oral administration of diethyl phthalate increased MDA, serum LH, FSH, SHBG, sperm abnormality, the percentage of spermatogonia, first spermatocyte, second spermatocyte, and spermatid in the testis, and decreased SOD, GSH, serum Ts and DHEA-SO_4_, testicular G6PD, 3βHSD, cholesterol, total protein, sperm count and motility, and sodium/potassium ATPase activity. Rat testis, epididymides, and prostates all shrink in response to diethyl phthalate, which explains this observation. Spermatogenic cells normally decline as a result of diethyl phthalate exposure, and the convoluted seminiferous tubule atrophy and denaturalization occurs. The conjunctive arrangements of cells have disappeared, and the spermatogenic and Sertoli cell configurations are notably asymmetrical [49], [50]. Diethyl phthalate-induced fetal Leydig cell aggregation eventually diminished, and this effect was associated with down-regulated Leydig cell biomarker mRNA levels and lower testicular Ts production. The formation of adult Leydig cells is thus disrupted by diethyl phthalate because it delays the involution of fetal Leydig cells [51]. As a result, diethyl phthalate dramatically decreased serum Ts levels. The male reproductive system relies principally on Ts, and spermatogenesis will finally cease if Ts are not present [52], [53]. This decrease in serum Ts can be explained by (1) the primary action of diethyl phthalate in the enzymatic stages of Ts biosynthesis, through cholesterol side chain cleavage and 3β-hydroxysteroid dehydrogenase-isomerase steps (which convert cholesterol to pregnenolone, where pregnenolone converts to progesterone, respectively), or (2) additional potential diethyl phthalate action sites, such as cholesterol side chain cleavage and 3β-hydroxysteroid dehydrogenase-isomerase steps, could be responsible for the decrease in Ts levels [54]. Due to testicular atrophy that occurs after exposure to diethyl phthalate, serum levels of Ts and DHEA-SO_4_ were decreased for an extended period of time [51]. Diethyl phthalate decreased 3βHSD levels in the testes and raised serum FSH and LH levels in this study. Numerous studies [55], [56], [57] have confirmed these results, showing that diethyl phthalate directly inhibits P450c17 activity in immature and precursor Leydig cells, along with P450 and/or 3βHSD activities. These effects may be ascribed to diethyl phthalate's pro-oxidant qualities. Ts, which is released by Leydig cells, when LH is present and controls spermatogenesis by binding to the androgen receptors on the germinal epithelium, was linked to the elevated FSH and LH values in diethyl phthalate exposure in this study. By targeting Sertoli cell receptors, FSH regulates spermatogenesis by inducing the development of many Sertoli cell components. Diethyl phthalate reduces the success of reproduction by interfering with the LH-stimulated Leydig cell's capacity to control spermatogenesis [58]. Because diethyl phthalate causes testicular dysfunction, which lowers testicular protein content, it lowered G6PD, 3βHSD, cholesterol, and total protein levels in this study. Diethyl phthalate reduced androgen secretion and synthesis, which in turn reduced cholesterol in the testis [59], [60]. Carrier proteins like SHBG, which aid in the transport of sex hormones across plasma, are among the primary determinants of the blood's concentration of unbound steroids [39], [61]. Because SHBG has a high binding affinity for Ts, which raises the amount of free and unbound albumin protein in the bloodstream, diethyl phthalate significantly increased SHBG while significantly decreasing Ts concentration [62]. The presence of carrier proteins in the blood alters the amount of steroid accessible to cells and impacts the dynamic balance between the percentages of bound and free steroids [41].

The percentage of spermatogonia, first spermatocytes, second spermatocytes, and spermatids in the testis rose after exposure to diethyl phthalate. Diethyl phthalate exposure increases sperm abnormalities while decreasing sperm motility and production. Diethyl phthalate interferes with the production of primary spermatocytes, round spermatids, and elongating spermatids, as well as the steroidogenesis and spermatogenic processes. A significant reduction in the number of sperm and disruption of testicular cells would probably follow significant degeneration of primary spermatocytes and round spermatids [63]. Exposure to diethyl phthalate causes the testis' proliferating index to increase and the number of viable sperm to decrease. This suggests that the number of sperm may have decreased because some spermatogenic cells may have been interrupted in their cycle. Thus, cell arrest led to cell death without the capacity to re-build spermatogenic cells, and the germ cells were doomed to die [64]. Diethyl phthalate raised MDA in the sperm, testis, and hypothalamus while decreasing SOD and GSH. The imbalance in the oxidative state caused by diethyl phthalate resulted in decreased sodium/potassium ATPase activity [65], [66]. This result is due to the fact that diethyl phthalate induces lipid peroxidation, which in turn produces free radicals, which cause oxidative damage and apoptosis. Diethyl phthalate inhibits the activities of G6PD and 6-phosphogluconate dehydrogenase (these two enzymes are involved in the oxidative phase pathway). As a result, less NADPH is available for glutathione reductase to use in maintaining the regeneration of reduced GSH [67]. Diethyl phthalate exposure causes sperm apoptosis and protein loss. This effect may be related to two mechanisms: (1) phthalate decreased functional Sertoli cell markers (Sox9, Sgp1, and Sgp2 mRNA). So, diethyl phthalate down-regulated Sox9 and disrupted the Pou4f1-Prnd gene network [68], and (2) a short-term increase in the p44/42 mitogen-activated protein kinase (MAPK) pathway was brought on by diethyl phthalate [69].

However, after receiving two oral doses of behenic acid, diethyl phthalate-treated rats showed a decrease in MDA, serum LH, FSH, SHBG, sperm abnormality, the percentage of spermatogonia, first spermatocyte, second spermatocyte, and spermatid in the testis, and a decrease in antioxidants such as SOD and GSH, serum Ts and DHEA-SO_4_, G6PD, 3βHSD, cholesterol, total protein, sperm count and motility, and sodium/potassium-ATPase activity. The higher dose of behenic acid had a more significant effect than the lower dose. The findings of numerous research studies, including Alqahtani et al. [70], concluded that behenic acid is a significant component of *Lepidium sativum s*eed oil and exhibits antibacterial, antioxidant, and anti-inflammatory properties, which is in line with this outcome. Additionally, Peng et al. [28] found that behenic acid has a positive impact on stroke prevention in the hypertensive population because it is inversely linked to acute ischemic stroke in hypertension patients. Furthermore, a higher behenic acid level is linked to a decreased incidence of pregnancy-induced hypertension in women, according to Li et al. [71]. Additionally, behenic acid has been linked to a lower incidence of type 2 diabetes mellitus, according to Huang et al. [72].

The mode of action of behenic acid on sperm to prevent sperm apoptosis and protein loss occurs in two ways: (1) By inhibiting the degeneration of Leydig and Sertoli cells, behenic acid increases sperm count, motility, and Ts and 3βHSD levels in the testis. (2) Behenic acid activates the hypothalamic-pituitary-gonadal axis of male rats, which results in a considerable increase in the Ts level [28]. Improvements in Ts and 3βHSD levels also generate a loop reaction that lowers LH and FSH levels [71].

As a whole, diethyl phthalate is a well-known hazardous chemical found in the environment that has dangerous effects on human and animal development and reproduction. Testicular oxidative damage is caused by diethyl phthalate by raising MDA levels and lowering antioxidant indicators, especially SOD and GSH. Also, diethyl phthalate disrupts the Sertoli cells in male reproductive organs. On the other hand, behenic acid guards against oxidative stress caused in the testicles by diethyl phthalates because the acid has antioxidant activity.

## 5 Conclusions

In order to protect the testis, hypothalamus, and sperm from oxidative stress brought on by diethyl phthalate, behenic acid reduced MDA and increased endogenous antioxidants, particularly SOD and GSH. This maintained normal sperm production and motility along with regular germ cell division. Moreover, behenic acid treatment safeguards normal male fertilization and stops sperm apoptosis and protein loss.

## Author contributions

Khaled Koriem selected the topic, performed the experimental steps, collected the outcome data and performed the statistical analysis for the study. **Mayar El-Masry** helped in the chromatography method of the study. Khaled Koriem and **Mayar El-Masry** participate in writing, reading, and approve the final manuscript.

## Funding

There is no funding for the study.

## CRediT authorship contribution statement

**Khaled M. M. Koriem**: Writing – review & editing, Supervision, Project administration, Methodology. **Mayar S. R. El-Masry**: Methodology.

## Declaration of Competing Interest

The authors declare that they have no known competing financial interests or personal relationships that could have appeared to influence the work reported in this paper.

## Data Availability

Data will be made available on request.
